# CRYAB suppresses ferroptosis and promotes osteogenic differentiation of human bone marrow stem cells via binding and stabilizing FTH1

**DOI:** 10.18632/aging.205851

**Published:** 2024-05-22

**Authors:** Bo Tian, Xiaolu Li, Weiyuan Li, Zhizhou Shi, Xu He, Shengyu Wang, Xun Zhu, Na Shi, Yan Li, Ping Wan, Chongtao Zhu

**Affiliations:** 1 Scientific Research Section, The First People’s Hospital of Yunnan Province, Kunming 650032, China; 2 Geriatric Department, The First People’s Hospital of Yunnan Province, Kunming 650032, China; 3 Medical School, Kunming University of Science and Technology, Kunming 650500, China; 4 Laser Medical Center, The First People’s Hospital of Yunnan Province, Kunming 650032, China; 5 The Affiliated Hospital of Kunming University of Science and Technology, Kunming 650032, China

**Keywords:** CRYAB, FTH1, ferroptosis, osteogenic differentiation, osteoporosis

## Abstract

Background: Bone formation and homeostasis are greatly dependent on the osteogenic differentiation of human bone marrow stem cells (BMSCs). Therefore, revealing the mechanisms underlying osteogenic differentiation of BMSCs will provide new candidate therapeutic targets for osteoporosis.

Methods: The osteogenic differentiation of BMSCs was measured by analyzing ALP activity and expression levels of osteogenic markers. Cellular Fe and ROS levels and cell viability were applied to evaluate the ferroptosis of BMSCs. qRT-PCR, Western blotting, and co-immunoprecipitation assays were harnessed to study the molecular mechanism.

Results: The mRNA level of CRYAB was decreased in the plasma of osteoporosis patients. Overexpression of CRYAB increased the expression of osteogenic markers including OCN, OPN, RUNX2, and COLI, and also augmented the ALP activity in BMSCs, on the contrary, knockdown of CRYAB had opposite effects. IP-MS technology identified CRYAB-interacted proteins and further found that CRYAB interacted with ferritin heavy chain 1 (FTH1) and maintained the stability of FTH1 via the proteasome mechanism. Mechanically, we unraveled that CRYAB regulated FTH1 protein stability in a lactylation-dependent manner. Knockdown of FTH1 suppressed the osteogenic differentiation of BMSCs, and increased the cellular Fe and ROS levels, and eventually promoted ferroptosis. Rescue experiments revealed that CRYAB suppressed ferroptosis and promoted osteogenic differentiation of BMSCs via regulating FTH1. The mRNA level of FTH1 was decreased in the plasma of osteoporosis patients.

Conclusions: Downregulation of CRYAB boosted FTH1 degradation and increased cellular Fe and ROS levels, and finally improved the ferroptosis and lessened the osteogenic differentiation of BMSCs.

## INTRODUCTION

Osteoporosis (OP) is a frequent bone disorder affecting 200 million people in the world, with the characteristics of bone microstructure alterations and bone loss [1–3]. The aging population and postmenopausal women are the high-risk populations of OP, and OP patients have a 40% risk of lifetime fracture which often occurs in the wrist, hip, or spine [4]. Therefore, uncovering the mechanisms underlying osteoporosis is quite important for developing new diagnostic and therapeutic methods for OP.

BMSCs could differentiate into several types of mature cells including osteoblasts which form new bone tissues, therefore, improving osteogenic differentiation of BMSCs is a promising method for OP therapy [5, 6]. Vildagliptin could enhance the osteogenic differentiation of BMSCs *in vitro* and further mitigate postmenopausal osteoporosis *in vivo* [7]. BushenHuoxue formula exhibited therapeutic effects on osteoporosis by improving osteogenic differentiation of BMSCs through regulating the Hedgehog pathway [8]. Kynurenic acid could alleviate osteoporosis in mice models and mechanically potentiate BMSCs osteogenesis by activating the Wnt/b-catenin signaling pathway [9]. Gentiopicroside was found to promote BMSC osteogenesis by affecting b-catenin/BMP2 pathway both *in vitro* and *in vivo* [10].

Ferroptosis, a newly-discovered programmed cell death, is different from necrosis, pyroptosis, apoptosis, etc. [11–14], and has the characteristics of reactive oxygen species (ROS) accumulation and iron-dependent lipid peroxidation [15, 16]. Ferroptosis is involved in the progression of several types of non-cancer diseases such as ischemia and reperfusion injury, viral infection, atherosclerosis, and so on [17, 18]. Quite importantly, a lot of studies have reported that ferroptosis participated in the development of osteoporosis. Cao et al*.* reported that ferroptosis potentiated bone absorption and attenuated bone formation, and finally led to osteoporosis [19]. High glucose suppresses the osteogenic function of osteoblasts by causing ferroptosis, and acid sphingomyelinase could improve the osteogenic function of osteoblasts via suppressing ferroptosis [20]. Mesenchymal stem cells-secreted exosomes could restore cartilage damage in OP through inhibiting ferroptosis via regulating the GOT1/CCR2/Nrf2/HO-1 signaling pathway [21]. FTH1, a key component of ferritin, suppresses lipid peroxidation and ferroptosis via regulating iron homeostasis [22]. The iron-enriched diet could lead to osteoporotic phenotypes in Hfe-KO mice with the reasons of osteoblast number decrease and FTH1 upregulation [23]. Nevertheless, the detailed roles and regulatory mechanisms of ferroptosis in the progression of osteoporosis are still largely unclear.

Crystallin Alpha B (CRYAB) is a molecular chaperone and plays crucial roles in the bioprocess of protein folding. CRYAB participates in many diseases and is considered as a potential therapeutic target. Mutations in CRYAB are correlated with autosomal dominant axonal Charcot-Marie-Tooth disease [24]. CRYAB showed cardioprotective effects by suppressing oxidative stress-caused apoptosis [25]. In infarcted rat hearts, transplantation of mature human induced pluripotent stem cell-derived cardiomyocytes could improve angiogenesis mainly through regulating CRYAB [26]. The chaperone activity and molecular dynamics of CRYAB were regulated by its phosphorylation. In retinal muller cells, the stability of CRYAB was regulated by its phosphorylation at Ser59 [27]. Intriguingly, during BMSCs osteogenesis but not chondrogenesis and adipogenesis, CRYAB was elevated [28, 29]. Functionally CRYAB interacted with and stabilized b-catenin, and eventually promoted osteogenic differentiation of BMSCs [29]. However, the role of CRYAB in ferroptosis during osteoporosis is still fairly unknown.

In the present study, we found CRYAB was decreased in osteoporosis, and further explored its roles and underlying mechanisms during osteogenic differentiation of BMSCs.

## MATERIALS AND METHODS

### Patients and samples

25 primary osteoporosis patients and 25 healthy people were enrolled in our study and samples were collected from First People’s Hospital of Yunnan Province (China) between March 2021 and November 2021. The osteoporosis was diagnosed by quantitative ultrasound (QUS) bone densitometry. 4 ml blood sample for each patient was collected and used to analyze the expression levels of CRYAB and FTH1.

### Cell culture and transfection

Human BMSCs were obtained from Mingzhoubio and cultured using DMEM with 10% FBS (Thermo Fisher Scientific, USA) at 37° C on 5% CO_2_. siRNAs and vectors were transfected using Lipofectamine 2000 (Thermo Fisher Scientific, USA), and CRYAB siRNA, FTH1 siRNA, CRYAB overexpression vector (CRYAB-OE), FTH1 overexpression vector (FTH1-OE) and negative control sequence were synthesized by GenePharma Co., Ltd. (China) (Table 1). Z-VAD-FMK (zFMK), necrostatin-1 (Nec-1), ferrostatin-1 (Fer-1), MG132, BafA1, sodium lactate (Nala), 2-Deoxy-D-glucose (2-DG) and oxamate were purchased from Selleck Chemicals (USA).

**Table 1:** Sequences of siRNAs and primers in the present study.

**NO.**	**Gene**	**Sequences (5’-3’)**
siRNAs		
1	siCRYAB	CCCUCUCACCAUUACUUCA
2	siFTH1	CCAUGUCUUACUACUUUGACC
3	NC	UUCUCCGAAC GUGUCACGU
Primers		
1	OCN forward	GGTGCAGCCTTTGTGTCCAA
	OCN reverse	CCTGAAAGCCGATGTGGTCA
2	OPN forward	AGCAGAATCTCCTAGCCCCA
	OPN reverse	ACGGCTGTCCCAATCAGAAG
3	RUNX2 forward	AACCCTTAATTTGCACTGGGTCA
	RUNX2 reverse	CAAATTCCAGCAATGTTTGTGCTAC
4	COLI forward	CGATGGATTCCAGTTCGAG
	COLI reverse	TAGGTGATGTTCTGGGAGGC
5	FTH1 forward	CCCCCATTTGTGTGACTTCAT
	FTH1 reverse	GCCCGAGGCTTAGCTTTCATT
6	CRYAB forward	CCTGAGTCCCTTCTACCTTCG
	CRYAB reverse	CACATCTCCCAACACCTTAACTT
7	GAPDH forward	TCATGGGTGTGAACCATGAGAA
	GAPDH reverse	GGCATGGACTGTGGTCATGAG

### RNA purification and qRT-PCR assay

RNeasy Mini Kit (Qiagen, Germany) and HiFiScript cDNA Synthesis Kit (CWBIO, China) were harnessed to perform RNA extraction and reverse transcription. The expression levels of CRYAB, OCN, OPN, RUNX2, COLI, and FTH1 were detected using SYBR™ Green PCR Master Mix (Thermo Fisher Scientific, USA). GAPDH and the formula 2^-ΔΔCt^ were used to normalize and calculate the relative expression of detected genes. The primer sequences are listed in Table 1.

### Cell viability, cellular Fe level, and ROS level detection

Cell Counting Kit-8 (Dojindo Laboratories, Japan) and QuantiChrom Iron Assay Kit (Bioassay Systems, USA) were harnessed to detect the cell viability and cellular Fe level according to each manufacturer’s instruction, respectively. Cellular ROS level was detected using the C11-BODIPY assay as described previously [30].

### Immunoprecipitation analysis

The interaction between CRYAB and FTH1 was analyzed by co-immunoprecipitation (co-IP) assay [31]. CRYAB antibodies (Proteintech, China, 15808-1-AP; Proteintech, 68001-1-Ig) and FTH1 antibodies (Cell Signaling Technology, USA, 4393) were used in the co-IP assay.

### KEGG and GO enrichment analyses

The KEGG (Kyoto Encyclopedia of Genes and Genomes) and GO (Gene Oncology) enrichment analyses were carried out using the DAVID database (https://david.ncifcrf.gov) [32].

### Western blotting assay

Western blotting assay was performed as previous study [33]. A RIPA buffer and a BCA Protein Assay Kit (Solarbio, China) were applied to extract total proteins and examine concentrations of samples. The information of antibodies was as follows: anti-OCN antibody (Proteintech, 23418-1-AP), OPN antibody (Proteintech, 22952-1-AP), RUNX2 (Abcam, UK, ab192256), COLI antibody (Proteintech, 14695-1-AP), CRYAB antibody (Proteintech, 15808-1-AP), FTH1 antibody (Cell Signaling Technology, 4393) and anti-GAPDH antibody (Abcam, ab8245).

### Statistical analysis

GraphPad Prism 9 (USA) was used to analyze the statistical significance of data (mean ± SD). Student’s t-test as well as ANOVA were carried out to analyze the data. *p* < 0.05 is defined as statistical significance.

## RESULTS

### CRYAB positively regulates the osteogenic differentiation of BMSCs

**Figure 1:**
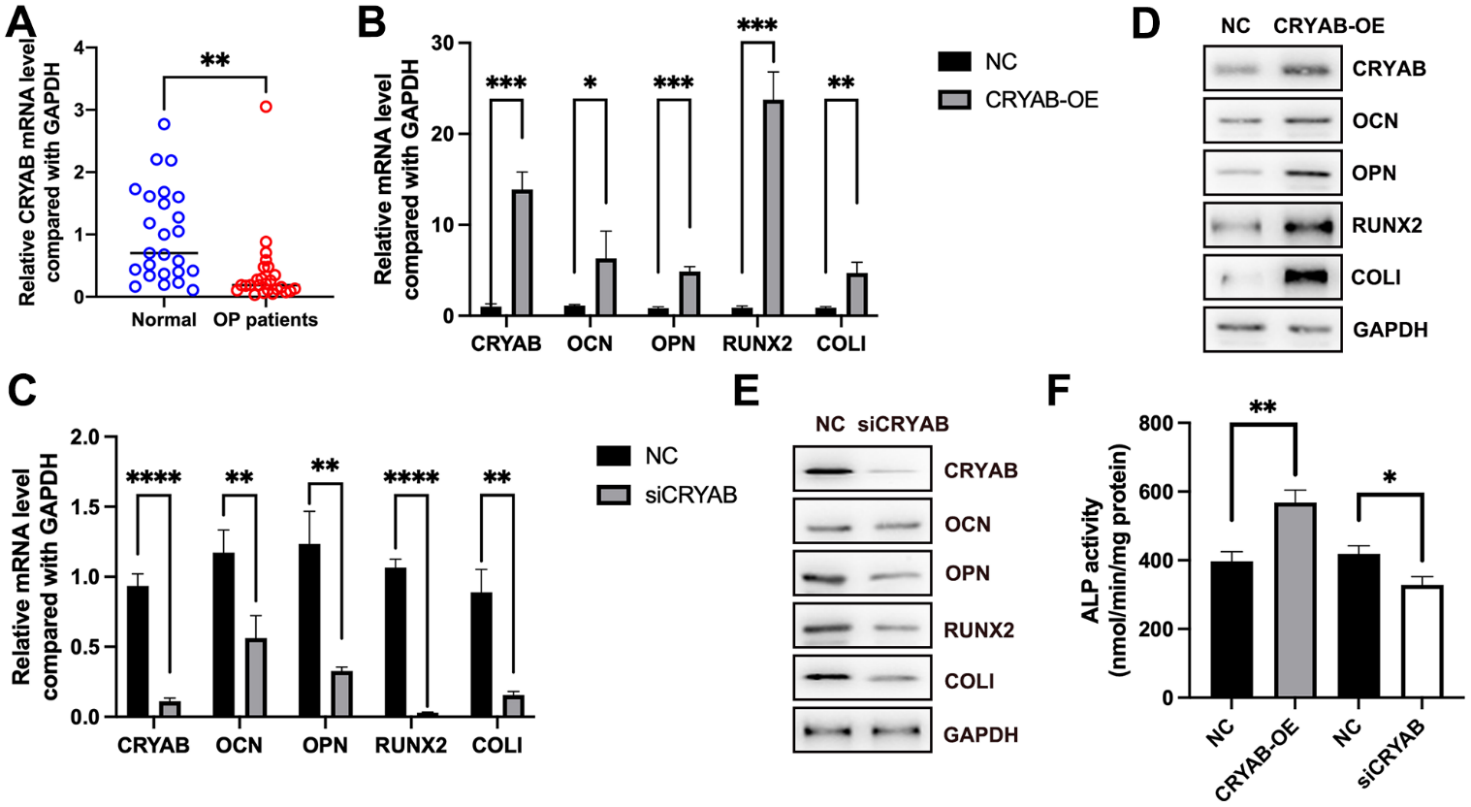
**CRYAB positively regulates the osteogenic differentiation of BMSCs.** (**A**) mRNA expression level of CRYAB in osteoporosis samples was detected using qRT-PCR. (**B**, **C**) mRNA expression levels of CRYAB, OCN, OPN, RUNX2, and COLI when CRYAB was overexpressed or silenced were detected using qRT-PCR (n=3). (**D**, **E**) Protein expression levels of CRYAB, OCN, OPN, RUNX2, and COLI when CRYAB was overexpressed or silenced were detected using the Western blotting method (n=3). (**F**) The activity of ALP was detected using an Alkaline Phosphatase Assay Kit (n=3). *: *p* < 0.05; **: *p* < 0.01; ***: *p* < 0.001; ****: *p* < 0.0001.

Using the qRT-PCR method, we found that the mRNA expression of CRYAB was decreased in osteoporosis (Figure 1A), indicative of the potential roles of CRYAB during osteoporosis progression. Importantly, when we overexpressed CRYAB in BMSCs, the mRNA expression levels of osteogenic marker genes including osteocalcin (OCN), osteopontin (OPN), runt-related transcription factor 2 (RUNX2), and type I collagen (COLI) were increased (Figure 1B). On the contrary, the knockdown of CRYAB reduced the mRNA expression levels of all these osteogenic marker genes (Figure 1C). Using the Western blotting method, we further revealed that overexpression of CRYAB upregulated OCN, OPN, RUNX2, and COLI, and in contrast, the silence of CRYAB downregulated OCN, OPN, RUNX2, and COLI at the protein level (Figure 1D, 1E). Alkaline Phosphatase (ALP) Assay Kit was applied to detect the activity of ALP, and overexpression of CRYAB increased the activity of ALP, and downregulation of CRYAB had the opposite effect on the ALP activity (Figure 1F). These findings suggested that CRYAB positively regulates the osteogenic differentiation of BMSCs.

### CRYAB interacts with FTH1 and regulates ferroptosis of BMSCs

**Figure 2:**
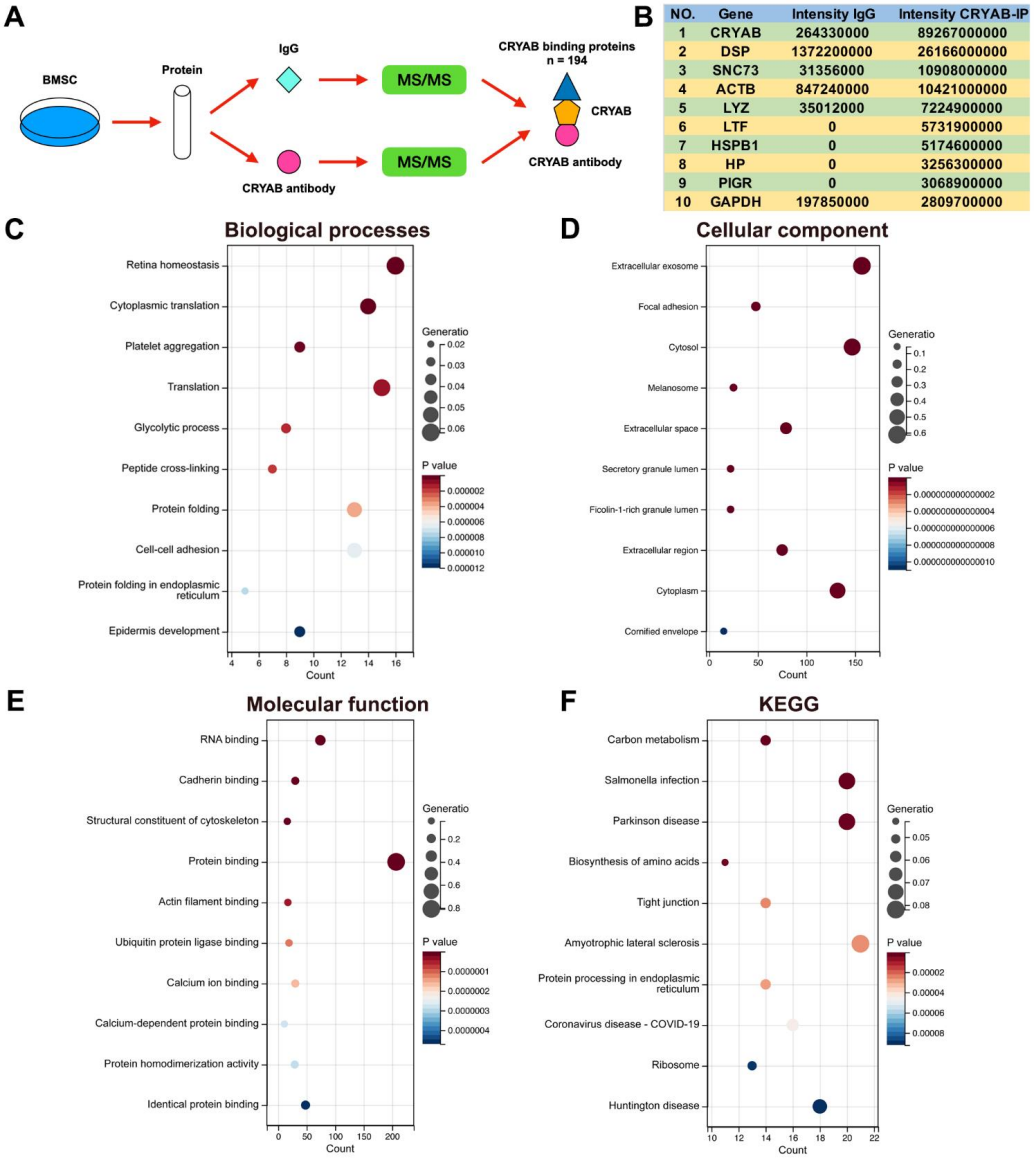
**Identification of CRYAB interacted proteins in BMSCs.** (**A**) IP-MS method was applied to identify the CRYAB interacted proteins in BMSCs. (**B**) The most abundantly identified top 10 CRYAB interacted proteins. (**C**–**F**) GO (biological process, cellular component, molecular function) and KEGG pathway were analyzed based on CRYAB interacted proteins.

Immunoprecipitation-mass spectrum (IP-MS) method was harnessed to identify the CRYAB interacted proteins in BMSCs (Figure 2A), and 194 proteins were selected (Fold change (Intensity BMSC-IP/Intensity BMSC-IgG) > 10, Supplementary Table 1). The most abundantly identified top 10 proteins were CRYAB, DSP, SNC73, ACTB, LYZ, LTF, HSPB1, HP, PIGR, and GAPDH (Figure 2B). GO and KEGG analyses were carried out based on CRYAB interacted proteins (n = 194). Biological process (BP) enrichment analysis indicated that CRYAB interacted proteins were correlated with retina homeostasis, cytoplasmic translation, platelet aggregation, translation, glycolytic process, and so on (Figure 2C). Cellular component (CC) enrichment analysis indicated that CRYAB interacted proteins were correlated with extracellular exosome, focal adhesion, cytosol, melanosome, extracellular space, and so on (Figure 2D). Molecular function (MF) enrichment analysis indicated that CRYAB interacted proteins were correlated with RNA binding, cadherin binding, structural constituent of cytoskeleton, protein binding, actin filament binding, and so on (Figure 2E). KEGG enrichment analysis indicated that CRYAB interacted proteins were correlated with carbon metabolism, salmonella infection, Parkinson disease, biosynthesis of amino acids, tight junction, and so on (Figure 2F).

**Figure 3:**
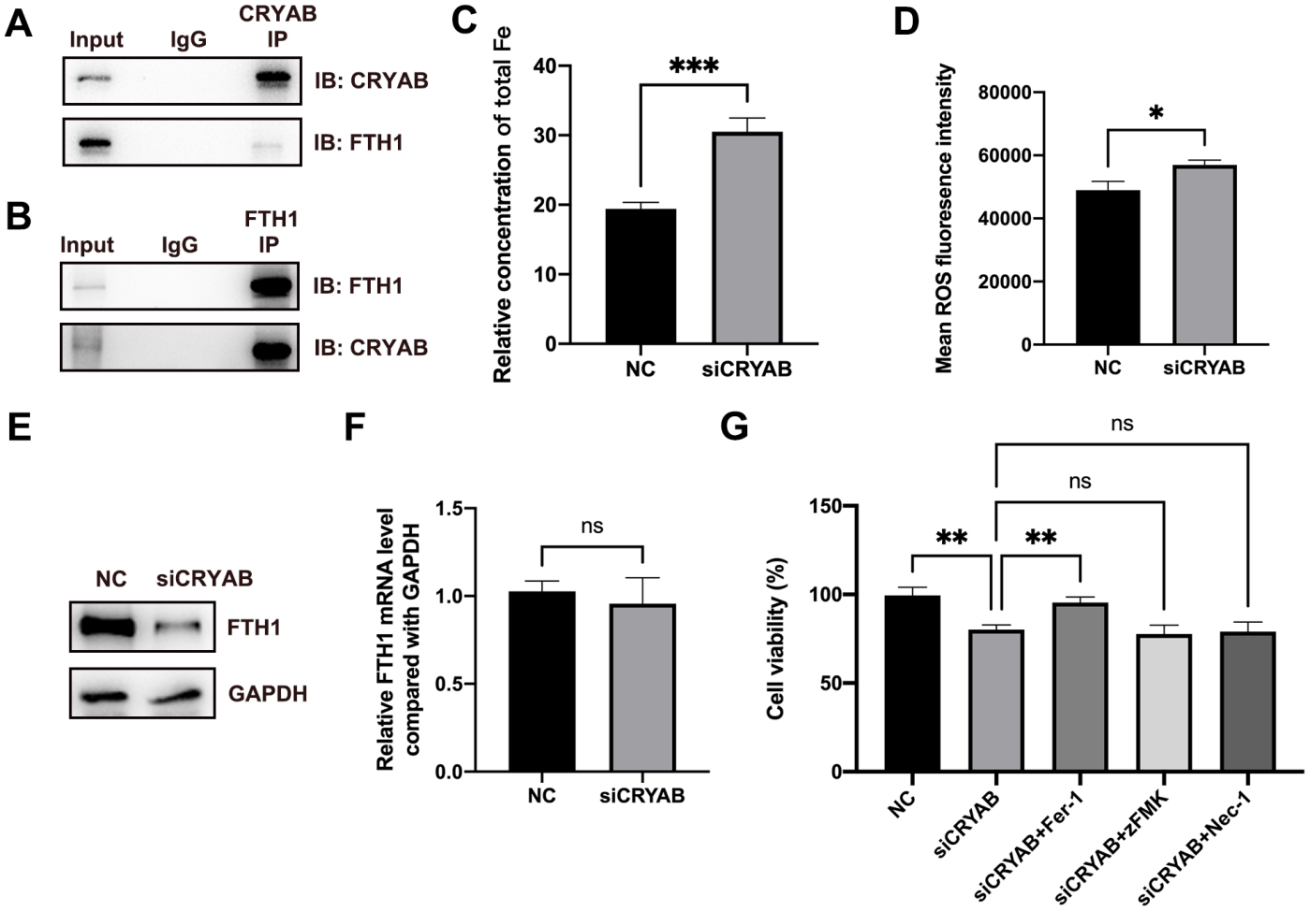
**CRYAB interacts with FTH1 and regulates the ferroptosis of BMSCs.** (**A**, **B**) Co-IP assay was used to detect the interaction between CRYAB and FTH1 (n=3). (**C**, **D**) Cellular Fe and ROS levels were detected after CRYAB knockdown in BMSCs (n=3). (**E**, **F**) The protein and mRNA levels of FTH1 were detected after CRYAB knockdown (n=3). (**G**) The cell viabilities were detected using CCK-8 assay (n=3). ns: no significance; *: *p* < 0.05; **: *p* < 0.01; ***: *p* < 0.001.

Very interestingly, FTH1, which played critical roles in ferroptosis via regulating cellular iron metabolism and iron storage [34] and was considered as a marker of ferroptosis [35], was identified as the CRYAB interacted protein (Supplementary Table 1). Given that ferroptosis participated in BMSCs osteogenesis [19–21], we selected FTH1 for further study. Co-immunoprecipitation (Co-IP) technology confirmed the interaction between CRYAB and FTH1 using both CRYAB antibody and FTH1 antibody for immunoprecipitation (Figure 3A, 3B). Importantly, we further found that the knockdown of CRYAB increased the cellular iron level and ROS level in BMSCs (Figure 3C, 3D). Western blotting assay showed that knockdown of CRYAB reduced the protein level of FTH1 but had no effect on the mRNA expression level, indicating that CRYAB regulated FTH1 at post-transcription level (Figure 3E, 3F). CCK-8 assay further revealed that silence of CRYAB declined the cell viability of BMSCs, and most importantly, the ferroptosis inhibitor (Ferrostatin-1, Fer-1) but not inhibitors of apoptosis (Z-VAD-FMK, zFMK) and necroptosis (Necrostatin-1, Nec-1) rescued the decrease of cell viability caused by CRYAB knockdown (Figure 3G). These results illustrated that CRYAB interacted with FTH1 and regulated ferroptosis of BMSCs.

### Knockdown of FTH1 promotes ferroptosis and lessens osteogenic differentiation of BMSCs

**Figure 4:**
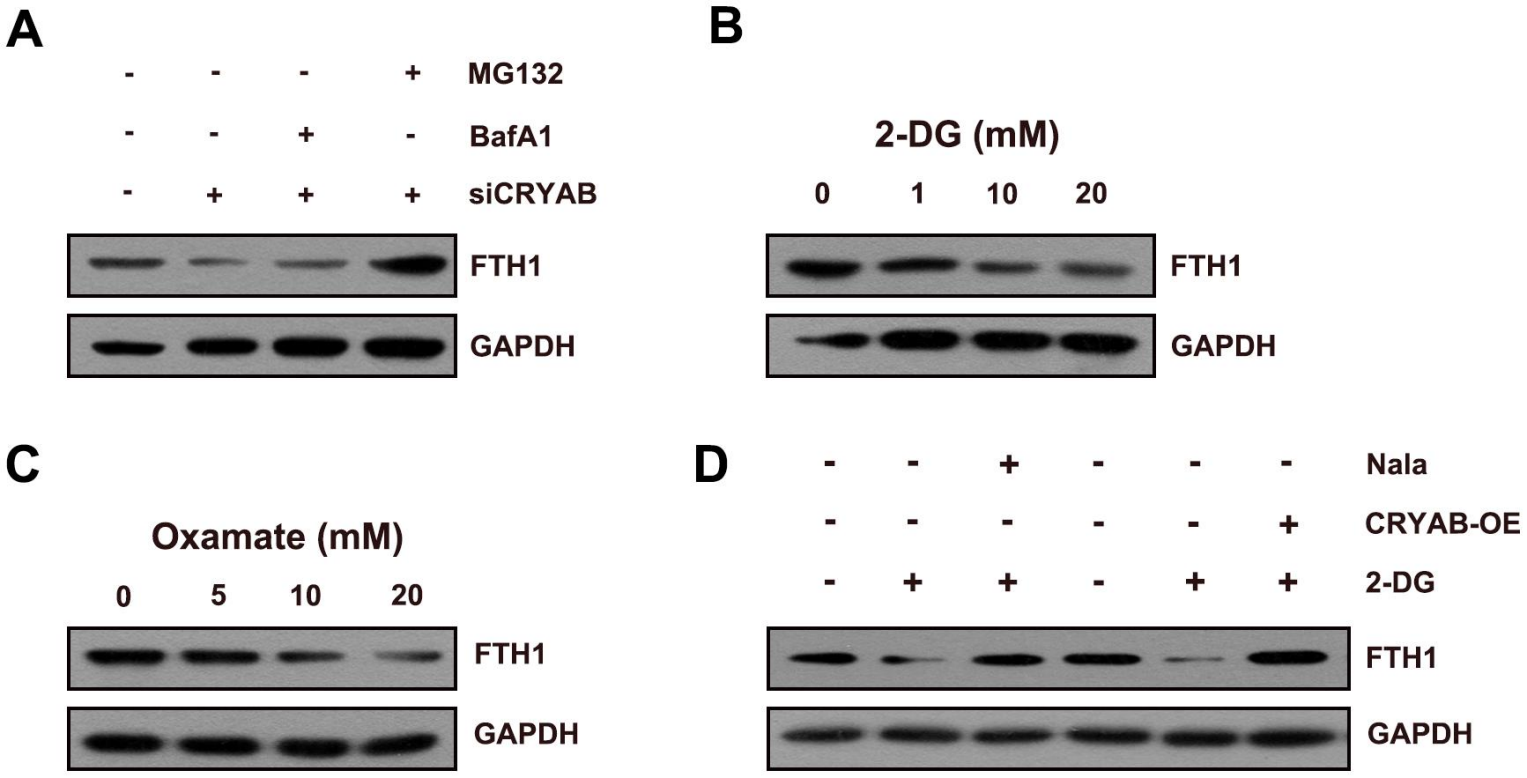
**CRYAB regulates the lactylation of FTH1 in BMSCs.** (**A**) Protein level of FTH1 was assessed after CRYAB knockdown and MG132/BafA1 treatment using Western blotting assay (n=3). (**B**, **C**) Protein level of FTH1 was detected after 2-DG or oxamate treatment (n=3). (**D**) Protein level of FTH1 was detected after 2-DG treatment (20 mM) and sodium lactate treatment (Nala, 20 mM) or CRYAB overexpression by Western blotting method (n=3).

Considering that FTH1 was regulated by CRYAB via the post-transcription mechanism, we speculated about whether CRYAB affected FTH1 through the degradation mechanism. Importantly, the proteasome inhibitor MG132 restored the protein level of FTH1 after CRYAB knockdown, yet the lysosomal inhibitor BafA1 had no effect on the protein level of FTH1 (Figure 4A). Then we further evaluated whether CRYAB regulated the protein stability of FTH1 via regulating lactylation mechanism. 2-DG and oxamate which were used to suppress the lactylation of global lactylation and histone lactylation could reduce the protein level of FTH1 in BMSCs (Figure 4B, 4C). Importantly, sodium lactate (Nala) and CRYAB overexpression could recover the 2-DG-induced downregulation of FTH1 respectively (Figure 4D).

These results confirmed that CRYAB affected FTH1 protein expression through the proteasome degradation mechanism.

**Figure 5:**
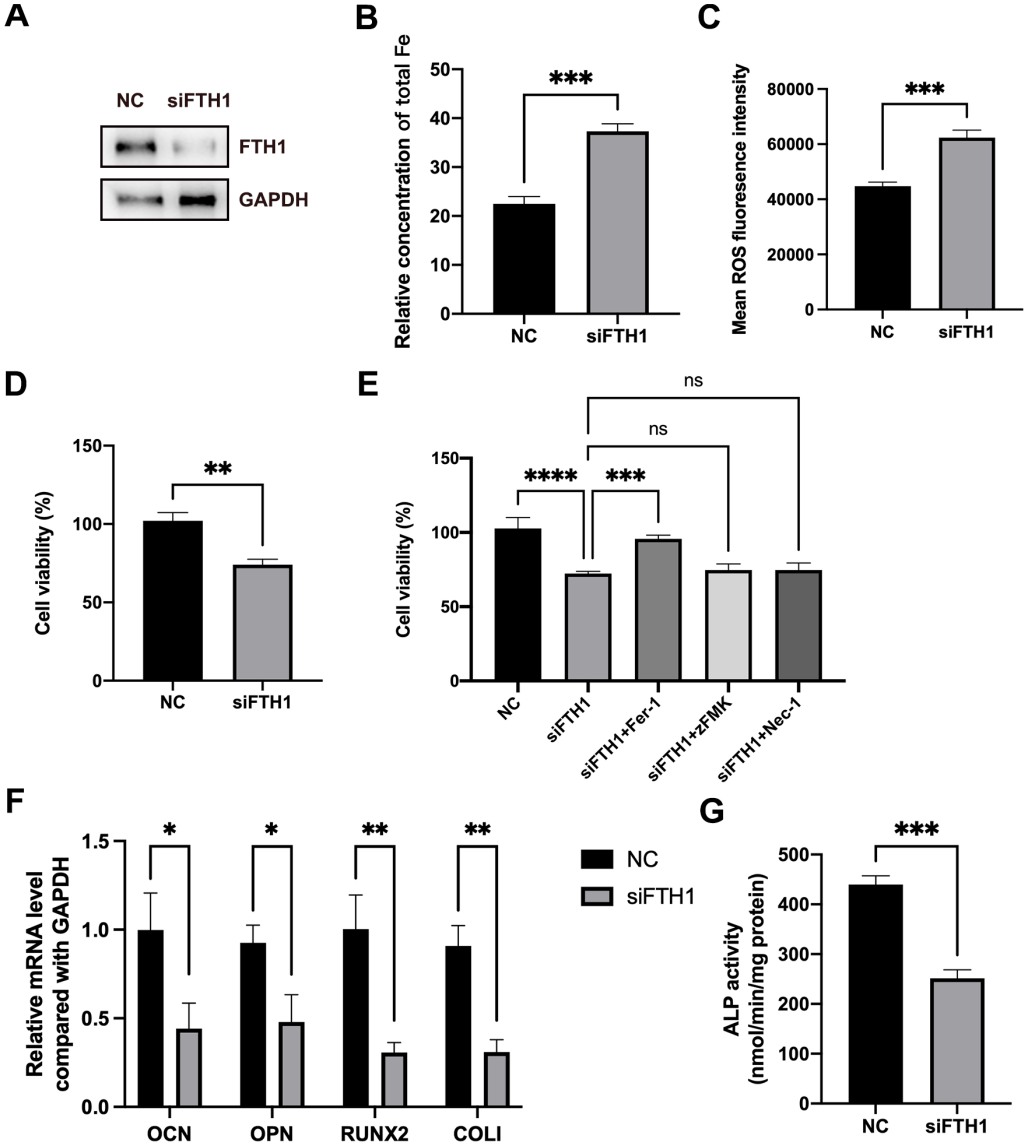
**Knockdown of FTH1 induces ferroptosis and suppresses osteogenic differentiation of BMSCs.** (**A**) The efficiency of FTH1 knockdown was validated using Western blotting method (n=3). (**B**, **C**) Cellular Fe and ROS levels were detected after FTH1 knockdown in BMSCs (n=3). (**D**, **E**) The cell viabilities were detected using CCK-8 assay (n=3). (**F**) mRNA expression levels of OCN, OPN, RUNX2, and COLI when FTH1 was silenced were detected using qRT-PCR (n=3). (**G**) The activity of ALP was detected using an Alkaline Phosphatase Assay Kit (n=3). ns: no significance; *: *p* < 0.05; **: *p* < 0.01; ***: *p* < 0.001; ****: *p* < 0.0001.

We further evaluated the effects of FTH1 silence on the ferroptosis and osteogenic differentiation of BMSCs. Knockdown of FTH1 significantly enhanced the cellular iron level and ROS level in BMSCs and also decreased the cell viability of BMSCs (Figure 5A–5D). Quite importantly, the ferroptosis inhibitor Fer-1 but not inhibitors of apoptosis zFMK and necroptosis Nec-1 rescued the decrease of cell viability caused by FTH1 knockdown (Figure 5E). Additionally, the silence of FTH1 reduced mRNA expression levels of osteogenic marker genes including OCN, OPN, RUNX2, and COLI, and also attenuated the activity of ALP in BMSCs (Figure 5F, 5G). These findings indicated that knockdown of FTH1 promoted ferroptosis and lessened osteogenic differentiation of BMSCs.

### CRYAB regulates ferroptosis and osteogenic differentiation of BMSCs in an FTH1-dependent manner

**Figure 6:**
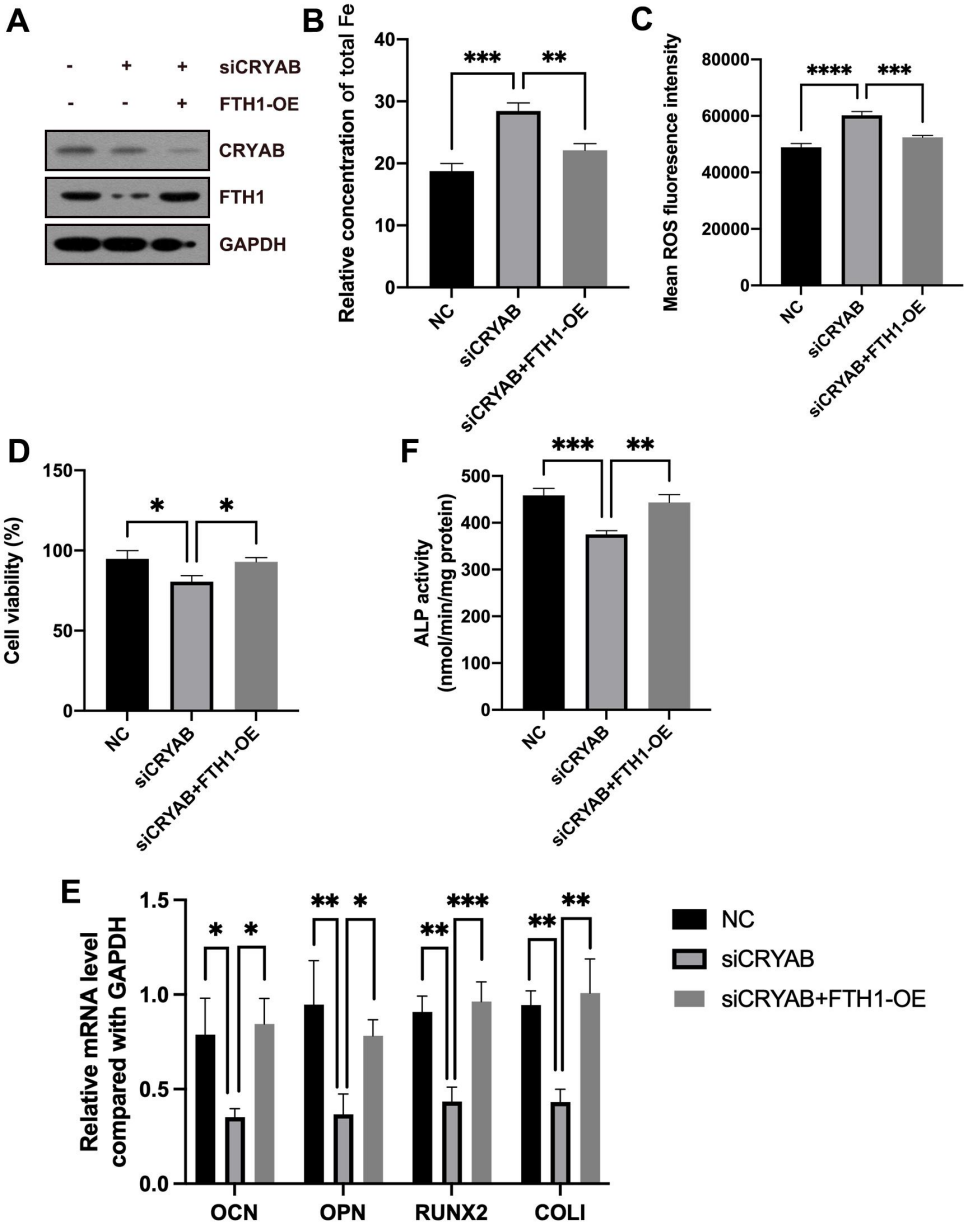
**CRYAB regulates ferroptosis and osteogenic differentiation of BMSCs in an FTH1-dependent manner.** (**A**) The protein levels of CRYAB and FTH1 were detected using a Western blotting assay (n=3). (**B**–**D**) Cellular Fe and ROS levels and cell viability were detected (n=3). (**E**) mRNA expression levels of OCN, OPN, RUNX2, and COLI were detected using qRT-PCR (n=3). (**F**) The activity of ALP was detected using an Alkaline Phosphatase Assay Kit (n=3). *: *p* < 0.05; **: *p* < 0.01; ***: *p* < 0.001; ****: *p* < 0.0001.

Rescue experiments were used to confirm the roles of FTH1 in CRYAB-affected ferroptosis and osteogenic differentiation of BMSCs. Compared with CRYAB-silenced BMSCs, the cellular Fe and ROS levels were significantly recovered in CRYAB-silenced and FTH1-overexpressed BMSCs (Figure 6A–6C). Exogenous expression of FTH1 also rescued the cell viability decrease led by CRYAB knockdown (Figure 6D). Quite importantly, overexpression of FTH1 restored the mRNA expression levels of osteogenic marker genes including OCN, OPN, RUNX2, and COLI, and the activity of ALP in BMSCs which were changed by CRYAB knockdown (Figure 6E, 6F). These results revealed that CRYAB regulated ferroptosis and osteogenic differentiation of BMSCs in an FTH1-dependent manner.

**Figure 7:**
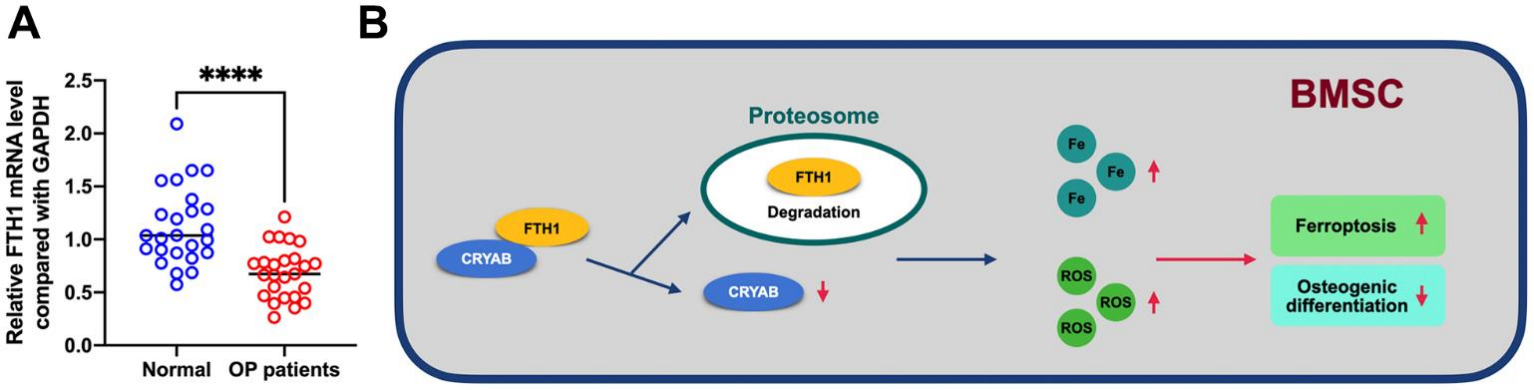
**FTH1 was decreased in osteoporosis.** (**A**) mRNA expression level of FTH1 in osteoporosis samples was detected using qRT-PCR. (**B**) Schematic illustration of the current study: downregulation of CRYAB boosted FTH1 degradation and increased cellular Fe and ROS levels, and finally improved the ferroptosis and lessened the osteogenic differentiation of BMSCs. ****: *p* < 0.0001.

In addition, we further evaluated the mRNA level of FTH1 in osteoporosis, and results showed that FTH1 declined in osteoporosis at the mRNA level (Figure 7A), providing evidence indicating that dysregulation of FTH1 was regulated at the protein level in a CRYAB-dependent manner and also at mRNA level in an unknown pathway. Collectively, the downregulation of CRYAB boosted FTH1 degradation and increased cellular Fe and ROS levels, and finally improved the ferroptosis and lessened the osteogenic differentiation of BMSCs (Figure 7B).

## DISCUSSION

The balance of new bone formation by osteoblasts and old bone resorption by osteoclasts affects bone remodeling. Osteogenic differentiation of BMSCs produces osteoblasts and affects bone formation. Therefore, studying and uncovering the mechanism underlying the osteogenic differentiation of BMSCs is quite important for the prevention and treatment of bone metabolism-related diseases including osteoporosis.

Up to now, several signaling pathways have been reported to participate in the regulation of osteogenic differentiation of BMSCs, and more and more methods have been developed to augment osteogenic differentiation. LncRNA HAGLR improved the osteogenic differentiation of BMSCs by affecting the miR-182-5p/Hoxa10 signaling pathway [36]. In mice models, BMSCs secreted exosomes, and then alleviated osteoporosis by regulating USP7/YAP1 and Wnt/β-catenin signaling pathways [37]. BMSCs secreted exosomes containing miR-182-5p-inhibitor and further promoted the bone regeneration of BMSCs [38]. Total flavonoids of Rhizoma drynariae were reported to promote the osteogenic differentiation of BMSCs via affecting ERR1/2-Gga1-TGF β-MAPK axis [39]. A traditional medicine codonopsis pilosula polysaccharides could activate β-catenin and further boost osteogenic differentiation of BMSCs [40]. Ma et al*.* reported that kynurenic acid could improve osteogenesis through activating the Wnt/β-catenin signaling pathway [9]. When Wnt signaling pathway was inhibited by QKI, an RNA-binding protein, the osteogenic differentiation was lessened [41]. Therefore, many signaling pathways especially the Wnt/β-catenin signaling pathway control the osteogenic differentiation of BMSCs.

A previous study has reported that during osteogenic differentiation of BMSCs, CRYAB was increased, and subsequent functional study further confirmed that CRYAB positively regulated osteogenic differentiation via Wnt/β-catenin signaling pathway [29]. Very importantly, our study unraveled that CRYAB interacted with and stabilized FTH1, sequentially suppressed ferroptosis of BMSCs via diminishing cellular Fe and ROS levels, and finally promoted osteogenic differentiation of BMSCs.

Ferroptosis is closely correlated with BMSCs osteogenesis, and blocking ferroptosis is a promising therapeutic strategy for osteoporosis. High-fat diet significantly induced ferroptosis and augmented bone loss [42]. Iron overload remarkably caused ferroptosis of osteoblasts and suppressed osteogenesis both *in vitro* and *in vivo* [43]. Interestingly, iron overload inactivated the Wnt signaling pathway and eventually dampened osteoblast differentiation [44]. Quercetin could suppress the ferroptosis of BMSCs by inhibiting the phosphorylation of PI3K, AKT, and mTOR [45]. Tocopherol could alleviate ferroptosis of BMSCs via regulating the PI3K/AKT/mTOR axis [46]. Engeletin could lessen Erastin-caused ferroptosis of BMSCs via Nrf2/Keap1 signaling pathway [47]. Ebselen could recover osteogenic suppression by blocking ferroptosis of BMSCs [48]. Blocking ferroptosis by SIRT6 could ameliorate bone formation and angiogenesis [49]. FTH1, the heavy subunit of ferritin, functions to store intracellular iron and regulate iron metabolism [34, 50]. Blocking FTH1 using baicalin or curcumenol effectively triggered ferroptosis in cancer cells and dampened tumor growth [51, 52]. Importantly, in Hfe-KO mice, an iron-enriched diet reduced osteoblast number, and upregulated FTH1, and caused osteoporotic phenotypes [23]. However, whether blocking FTH1 could mitigate BMSC osteogenesis and osteoporosis *in vivo* is still unclear.

Our study revealed that CRYAB bound with FTH1 and stabilized FTH1 protein via the proteasome mechanism and then affected cellular Fe and ROS levels and ferroptosis of BMSCs. Although our study revealed that iron level in BMSCs was regulated by CRYAB-FTH1 complex, whether the Wnt/β-catenin signaling pathway was involved in CRYAB-FTH1 complex-regulated, ferroptosis and osteogenic differentiation of BMSCs is still needed to be studied in future.

## Supplementary Material

Supplementary Table 1
