# Supplementary material for: CRYAB suppresses ferroptosis and promotes osteogenic differentiation of human bone marrow stem cells via binding and stabilizing FTH1
Source: Aging (Albany NY). 2024 May 22;16(10):8965–79. doi: 10.18632/aging.205851 (PMC11164484; doi:10.18632/aging.205851)
Supplement: Supplementary Table 1 [file aging-16-205851-s001.docx]

Supplementary Table 1. Identification of CRYAB-interacted proteins in BMSCs using IP-MS technology.

| NO. | Gene | Intensity BMSC-IgG | Intensity BMSC-IP |
| --- | --- | --- | --- |
| 1 | CRYAB | 264330000 | 89267000000 |
| 2 | DSP | 1372200000 | 26166000000 |
| 3 | SNC73 | 31356000 | 10908000000 |
| 4 | ACTB | 847240000 | 10421000000 |
| 5 | LYZ | 35012000 | 7224900000 |
| 6 | LTF | 0 | 5731900000 |
| 7 | HSPB1 | 0 | 5174600000 |
| 8 | HP | 0 | 3256300000 |
| 9 | PIGR | 0 | 3068900000 |
| 10 | GAPDH | 197850000 | 2809700000 |
| 11 | LCN1 | 22084000 | 2412400000 |
| 12 | PKM | 115280000 | 2025500000 |
| 13 | TBC1D10C | 0 | 1874700000 |
| 14 | AZGP1 | 0 | 1683000000 |
| 15 | PRDX1 | 94696000 | 1596400000 |
| 16 | CASP14 | 34289000 | 1582800000 |
| 17 | S100A9 | 75959000 | 1527900000 |
| 18 | JCHAIN | 27898000 | 1426300000 |
| 19 | SERPINB3 | 31777000 | 1374600000 |
| 20 | BLMH | 39722000 | 1345100000 |
| 21 | DKFZp686M08189 | 0 | 1100200000 |
| 22 | CALML5 | 9122300 | 1007700000 |
| 23 | ENO1 | 36448000 | 1007100000 |
| 24 | ACTA2 | 0 | 961110000 |
| 25 | HAL | 8352900 | 866320000 |
| 26 | FABP5 | 42996000 | 852390000 |
| 27 | TPI1 | 20344000 | 791340000 |
| 28 | SOD2 | 52149000 | 791230000 |
| 29 | P4HB | 7650500 | 776710000 |
| 30 | TUBB | 0 | 757200000 |
| 31 | ARG1 | 15629000 | 719460000 |
| 32 | LOR | 44838000 | 711060000 |
| 33 | EEF2 | 7300900 | 704670000 |
| 34 | CTSD | 14160000 | 689240000 |
| 35 | SBSN | 5390100 | 636800000 |
| 36 | TRIM21 | 0 | 633920000 |
| 37 | PFN1 | 4784900 | 629100000 |
| 38 | LDHA | 39762000 | 616200000 |
| 39 | TXN | 0 | 598540000 |
| 40 | HSPA8 | 8255400 | 598030000 |
| 41 | HSPA9 | 0 | 587830000 |
| 42 | ALDOA | 0 | 585420000 |
| 43 | PPIB | 35448000 | 550760000 |
| 44 | GSTP1 | 0 | 535530000 |
| 45 | YWHAZ | 0 | 502550000 |
| 46 | HBB | 25334000 | 501700000 |
| 47 | LGALS1 | 0 | 489830000 |
| 48 | GGCT | 6196300 | 488450000 |
| 49 | LMNA | 0 | 485030000 |
| 50 | PRICKLE2 | 0 | 437680000 |
| 51 | ASPRV1 | 0 | 423390000 |
| 52 | GSDMA | 19276000 | 423350000 |
| 53 | CALR | 0 | 417740000 |
| 54 | TGM3 | 14207000 | 398930000 |
| 55 | TPM4 | 8559800 | 389590000 |
| 56 | PIP | 0 | 362340000 |
| 57 | DEFA3 | 0 | 342310000 |
| 58 | PDIA3 | 6029000 | 334800000 |
| 59 | HSP90B1 | 0 | 329910000 |
| 60 | POF1B | 28570000 | 295940000 |
| 61 | KRT6B | 0 | 294210000 |
| 62 | ACTN4 | 0 | 273460000 |
| 63 | WDR11 | 0 | 269690000 |
| 64 | S100A7 | 0 | 258480000 |
| 65 | RPS3 | 0 | 241710000 |
| 66 | LGALS7B | 11150000 | 231350000 |
| 67 | S100A16 | 7781600 | 230850000 |
| 68 | RAB1B | 13902000 | 226550000 |
| 69 | MYL6 | 0 | 218290000 |
| 70 | CTSB | 0 | 209820000 |
| 71 | SERPINB12 | 12322000 | 203830000 |
| 72 | HBB | 0 | 203300000 |
| 73 | RTN4 | 14057000 | 202830000 |
| 74 | IDE | 0 | 199060000 |
| 75 | QPRT | 0 | 196770000 |
| 76 | B2M | 0 | 189690000 |
| 77 | ATP5F1B | 13395000 | 186700000 |
| 78 | S100A11 | 10992000 | 185530000 |
| 79 | AKR1B1 | 10827000 | 170590000 |
| 80 | ANXA5 | 11609000 | 168450000 |
| 81 | HSPD1 | 11859000 | 162020000 |
| 82 | CALM3 | 0 | 159540000 |
| 83 | ALOX12B | 0 | 152470000 |
| 84 | RAB6B | 0 | 150490000 |
| 85 | NME1 | 0 | 147600000 |
| 86 | CAT | 8462500 | 140630000 |
| 87 | S100A14 | 11360000 | 138300000 |
| 88 | TF | 0 | 132490000 |
| 89 | EPFP1 | 0 | 127800000 |
| 90 | SCGB2A1 | 0 | 125580000 |
| 91 | HNRPH3 | 0 | 125180000 |
| 92 | CCDC159 | 0 | 123110000 |
| 93 | MSN | 4617200 | 120950000 |
| 94 | EWSR1 | 7809500 | 117530000 |
| 95 | PON1 | 0 | 114950000 |
| 96 | EPPK1 | 0 | 108930000 |
| 97 | PGK1 | 0 | 108900000 |
| 98 | IFITM2 | 0 | 107910000 |
| 99 | EIF4A2 | 8609400 | 107010000 |
| 100 | ISG15 | 0 | 105710000 |
| 101 | RPS13 | 0 | 102710000 |
| 102 | TALDO1 | 0 | 97993000 |
| 103 | MDH2 | 0 | 92452000 |
| 104 | CANX | 0 | 89865000 |
| 105 | ANXA1 | 2207700 | 89708000 |
| 106 | LACRT | 0 | 89165000 |
| 107 | ACTG1 | 5401300 | 85732000 |
| 108 | C4B | 0 | 82511000 |
| 109 | IDH1 | 5708800 | 81395000 |
| 110 | RPS4X | 0 | 79931000 |
| 111 | VDAC2 | 0 | 78608000 |
| 112 | PSMA8 | 0 | 76812000 |
| 113 | GLRX | 0 | 74776000 |
| 114 | VCP | 0 | 71066000 |
| 115 | RPS19 | 0 | 65098000 |
| 116 | ARPC4 | 0 | 64479000 |
| 117 | ORM2 | 0 | 64323000 |
| 118 | TUBB2C | 0 | 61960000 |
| 119 | LTF | 0 | 60603000 |
| 120 | VDAC1 | 0 | 59624000 |
| 121 | RAB11A | 0 | 59402000 |
| 122 | DNCL1 | 0 | 59320000 |
| 123 | PRDX4 | 0 | 58458000 |
| 124 | CLIC1 | 0 | 58217000 |
| 125 | SLC25A3 | 0 | 57756000 |
| 126 | NFE2L2 | 0 | 56994000 |
| 127 | RAB5A | 0 | 56567000 |
| 128 | TAGLN2 | 0 | 55665000 |
| 129 | ATP5PO | 0 | 55428000 |
| 130 | APOA1 | 0 | 55428000 |
| 131 | PRDX6 | 0 | 54730000 |
| 132 | GDI2 | 0 | 53682000 |
| 133 | CD44 | 0 | 52240000 |
| 134 | RPLP0 | 0 | 52145000 |
| 135 | KLK7 | 0 | 52093000 |
| 136 | PRR4 | 0 | 51835000 |
| 137 | PDIA6 | 0 | 49731000 |
| 138 | ECM1 | 0 | 47807000 |
| 139 | SOD1 | 0 | 45344000 |
| 140 | ZG16B | 0 | 45050000 |
| 141 | VAT1 | 0 | 44231000 |
| 142 | PLS3 | 0 | 43284000 |
| 143 | RACK1 | 0 | 42966000 |
| 144 | RCN1 | 0 | 42702000 |
| 145 | MARCKS | 0 | 42339000 |
| 146 | RPS5 | 3950700 | 41302000 |
| 147 | PSMA2 | 0 | 41284000 |
| 148 | FTH1 | 3320000 | 41219000 |
| 149 | RPL11 | 0 | 41168000 |
| 150 | RAN | 0 | 39776000 |
| 151 | ERH | 0 | 39369000 |
| 152 | PSMA3 | 0 | 39195000 |
| 153 | PSMB6 | 0 | 37915000 |
| 154 | CPA4 | 0 | 37634000 |
| 155 | RPLP1 | 0 | 37518000 |
| 156 | TAF15 | 0 | 36197000 |
| 157 | TIMM13 | 0 | 34050000 |
| 158 | ITGB1 | 0 | 33859000 |
| 159 | ARPC5 | 0 | 33660000 |
| 160 | HEL2 | 0 | 32563000 |
| 161 | GOT2 | 0 | 29643000 |
| 162 | FGF7 | 0 | 29456000 |
| 163 | SLC25A11 | 0 | 27797000 |
| 164 | IQGAP1 | 0 | 27264000 |
| 165 | PSAPL1 | 0 | 26957000 |
| 166 | PRKCSH | 0 | 26809000 |
| 167 | LEPR | 0 | 26041000 |
| 168 | YWHAB | 0 | 25528000 |
| 169 | ARPC1B | 0 | 23112000 |
| 170 | ATP5MG | 0 | 22758000 |
| 171 | CS | 0 | 22611000 |
| 172 | ASAH1 | 0 | 22294000 |
| 173 | GSTO1 | 0 | 21077000 |
| 174 | TUBAL3 | 0 | 20455000 |
| 175 | SSR4 | 0 | 19942000 |
| 176 | SLPI | 0 | 19213000 |
| 177 | HMGB1 | 0 | 19043000 |
| 178 | IVL | 0 | 18848000 |
| 179 | RPN1 | 0 | 18579000 |
| 180 | TAGLN | 0 | 18434000 |
| 181 | CLTC | 0 | 18286000 |
| 182 | TLN1 | 0 | 17530000 |
| 183 | HSD17B4 | 0 | 17217000 |
| 184 | ATP5PB | 0 | 16785000 |
| 185 | GPI | 0 | 16568000 |
| 186 | TUBB3 | 0 | 15862000 |
| 187 | DKFZp779L2418 | 0 | 15315000 |
| 188 | CRCT1 | 0 | 15105000 |
| 189 | FHIP1B | 0 | 12380000 |
| 190 | CPNE4 | 0 | 11788000 |
| 191 | ANXA6 | 0 | 9881600 |
| 192 | UTS2R | 0 | 8798400 |
| 193 | MX1 | 0 | 7116800 |
| 194 | TYMP | 0 | 3969800 |
